# Approaches to integrated monitoring for environmental health impact assessment

**DOI:** 10.1186/1476-069X-11-88

**Published:** 2012-11-21

**Authors:** Hai-Ying Liu, Alena Bartonova, Mathilde Pascal, Roel Smolders, Erik Skjetne, Maria Dusinska

**Affiliations:** 1 NILU - Norwegian Institute for Air Research, Instituttveien 18, 2027, Kjeller, Norway; 2 InVS - French Institute for Public Health Surveillance, 12, rue du Val d’Osne, 94415, Saint-Maurice cedex, France; 3 VITO - Flemish Institute for Technological Research, Boeretang 200, 2400, Mol, Belgium; 4 Statoil - Statoil Research Center, Arkitekt Ebbells Veg 10, Rotvoll, 7005, Trondheim, Norway

## Abstract

Although Integrated Environmental Health Monitoring (IEHM) is considered an essential tool to better understand complex environmental health issues, there is no consensus on how to develop such a programme. We reviewed four existing frameworks and eight monitoring programmes in the area of environmental health. We identified the DPSEEA (Driving Force-Pressure-State-Exposure-Effect-Action) framework as most suitable for developing an IEHM programme for environmental health impact assessment. Our review showed that most of the existing monitoring programmes have been designed for specific purposes, resulting in narrow scope and limited number of parameters. This therefore limits their relevance for studying complex environmental health topics. Other challenges include limited spatial and temporal data availability, limited development of data sharing mechanisms, heterogeneous data quality, a lack of adequate methodologies to link disparate data sources, and low level of interdisciplinary cooperation. To overcome some of these challenges, we propose a DPSEEA-based conceptual framework for an IEHM programme that would enable monitoring and measuring the impact of environmental changes on human health. We define IEHM as ‘a systemic process to measure, analyse and interpret the state and changes of natural-eco-anthropogenic systems and its related health impact over time at the same location with causative explanations across the various compartments of the cause-effect chain’. We develop a structural work process to integrate information that is based on existing environmental health monitoring programmes. Such a framework allows the development of combined monitoring systems that exhibit a large degree of compatibility between countries and regions.

## Background

Integrated Environmental Health Monitoring (IEHM) is essential for identifying key stressors on the environment, to assess the state of the environment, and to evaluate the health impact of environmental changes [1]. Currently, there is no agreed definition of IEHM. The European Union (EU) funded project INTARESE (Integrated Assessment of Health Risks of Environmental Stressors in Europe) explored ways of linking and integrating various information sources and technologies to provide a more unified approach to IEHM.

The aim of IEHM is to provide unbiased data of appropriate quality and quantity for IEHIA (Integrated environmental health impact assessment), defined as ‘an inclusive and, as far as feasible, comprehensive assessment of the risks to, and impacts on, human health as a result either of exposures to a defined set of environmental hazards or of the effects of policies or other interventions that operate via the ambient or living environment’ [2,3]. Accordingly, information is required about the nature, the causes, and the inter-linkages between existing environmental health risks. In the past, research studies and policy actions often addressed single-pollutant and single-effect relationships, and there was no integration of data on exposure and impact of environmental changes on human health [4]. Recently however, efforts to understand the links between multiple stressors and multiple health effects are rapidly increasing. Experience has shown that integrated studies are often limited by the lack of data, or by the fact that different data collection systems have different goals and are, therefore, not easily combined.

The key issue for IEHM is to consider monitoring as a tool to measure, analyse and interpret the impact of environmental changes on human health, to support more effective decision-making. Ideally, a systematic, iterative process based on the knowledge of the cause-effect chain is needed to describe the interconnected environment and health (E&H) issues. The complexity of E&H problems and the interaction of multiple parameters at each level of organization and scale [5] pose challenges to the design of an IEHM programme and require innovative ways to make better use of existing data, including technological solutions to data integration.

In generally comprehensive analyses, it is often difficult to incorporate data on health effects due to their varying quality or lack of representativeness. An inter-disciplinary IEHM programme based on a cause-effect chain approach to the natural-eco-anthropogenic system elements would allow such analysis [6,7].

In Europe, a number of initiatives to link E&H information already exist: the World Health Organisation (WHO) ‘Health for All’ initiatives; the WHO European Centre for Environment and Health (ECEH) indicator work; the European Integrated Environment and Health Monitoring and Response System (EIEHMRS); and the European initiatives European Community Health Indicators (ECHI). These systems aggregate data from heterogeneous sources but do not share strategies or objectives.

This paper develops a framework for an IEHM programme to monitor and measure the impact of environmental changes on human health, and, informed by existing monitoring programmes, proposes a structured work process that allows the gathering of data and information. We also review and discuss the main challenges faced by an IEHM programme.

### Frameworks

An IEHM programme should be based on a structured framework to enable simultaneous monitoring and interpretation [8]. A framework provides a systematic approach that helps identifying links or relationships between the environment and human health to interpret complex E&H issues. Indeed, the main role of a framework is to organize the concepts, ideas, and notions of an activity meaningfully [9], in order to recognize and interpret complex links between the elements of the activity [10].

Robust frameworks have [8]:

• Conceptual clarity and scope – ensuring that the framework covers all key concepts and includes logical and plausible links.

• Flexibility – to allow for consideration of the issue at any stage or component of the framework.

• Balance – the framework accommodates issues with an environmental or health emphasis equally well.

• Usability – the framework lends itself to a viable methodology for developing suitable indicators.

Developing an IEHM programme requires identifying links between environmental factors and human health outcomes. This allows the development of response strategies to changes in the environment. A framework needs to allow flexible extensions to be able to include indicators describing interactions in the natural-eco-anthropogenic systems. Therefore, a framework that groups indicators into determinants (e.g., physical, social, economic, environmental and behavioural health determinants) and outcomes is useful. Such an approach is much more informative than simply listing indicators.

Various frameworks have been developed in the areas of environment, health, and environmental health. Here we review four frameworks with respect to different attributes for developing an IEHM programme to measure and monitor the impacts of environmental change on human health [8] (Table 1):

• Driving Forces-Pressures-State-Impacts-Responses (DPSIR)

Driving Force-Pressure-State-Exposure-Effect-Action (DPSEEA)

• Multiple Exposures Multiple Effects (MEME)

• Integrated Environmental Health Impact Assessment

**Table 1:** **Attributes of the frameworks** (**see text for abbreviations**)

**Framework attributes**	**Frameworks**			
	**DPSIR**	**DPSEEA**	**MEME**	**IEHIA**
Designed for indicators	Yes	Yes	Yes	Yes
Includes environment & health components	Yes	Yes	Yes	Yes
Utilizes causal chain approach	Yes	Yes	Yes	Yes
Describes distal causal factors in detail	Yes	Yes	No	Yes
Explicitly includes exposure route	No	Yes	Yes	Yes
Explicitly includes actions/interventions	Yes	Yes	Yes	Yes
Explicitly includes multiple entry points for actions/interventions	Yes	Yes	Yes	No
Can be adapted to measure & monitor the impacts of environmental change on human health	No	Yes	Yes	Yes

Attributes refers to historically used and potential attributes of a framework.

The DPSIR framework (Additional file 1) adopted by the European Environmental Agency (EEA) and United Nations Environmental Programme (UNEP) to describe interactions between society and the environment, is an extension of the Pressure-State-Response (PSR) and the Driving force-State-Response (DSR) models [11,12]. **Driving forces** are the socio-economic and socio-cultural forces driving human activities, which increase or mitigate pressures on the environment. **Pressures** are the stresses that human activities place on the environment. **State**, or state of the environment, is the condition of the environment. **Impacts** are the effect of environmental degradation on human welfare. **Responses** refer to the responses by society to the environmental situation. The DPSIR is primarily focused on the environment and was designed to develop environmental indicators (Table 1). The DPSIR also considers the effects of the environment on human health, although they are not the primary focus of the framework [8,13].

The major limitations of DPSIR are: (i) focusing on the environment and development of environmental indicators, and impacts from environmental degradation; (ii) focusing on anthropogenic drivers and pressures, omitting the impacts from natural disturbances; and (iii) not differentiating dynamic processes that occur between exposure and effects. Due to the limited description of the exposure route (Table 1), the DPSIR cannot identify within this route multiple entry points for action. A further criticism is DPSIR’s tendency to portray the interaction between human activity and the environment as a unidirectional and linear relationship [11-17]. Due to these limitations, the DPSIR is considered unsuitable for describing the linkages between the E&H in detail, and thus cannot provide the guidance required to develop an IEHM programme to measure and monitor the impact of environmental changes on human health.

The DPSEEA framework (Additional file 2) was developed by the WHO [18] to support the development of E&H indicators (Table 1). **Driving force** (anthropogenic) incorporates factors that motivate and push the environmental process involved. **Pressure** (on the environment) is normally expressed through human occupation or exploitation of the environment. **State** presents the status of the environment. **Exposure** (of humans) takes place when humans are exposed to environmental conditions. **Effect** (in humans) indicates health effects from exposure to the environmental hazard. **Action** indicates policies or interventions aimed at reducing or avoiding health effects; they can be included at any point in the framework (Table 1).

Compared to DPSIR, the advantages of DPSEEA are: (i) it recognizes the links between exposure and health effects [18] (Table 1); (ii) it allows for several entry points in the cause-effect chain, linked to various levels of action that can be undertaken to reduce E&H impacts (Table 1, Additional file 2); (iii) it extends the concept of driving forces to more remote, contextual factors such as social and economic development [9,19-21], and (iv) it is flexible [22] and can be adapted and modified according to changing requirements and circumstances [8].

DPSEEA addresses more anthropogenic indicators, but is less effective for representing natural and physical risks, so the complex interactions between natural and human systems are not well represented [9,20,21]. There are also limitations if the framework is applied in a linear form [9,14-17,19,21-25]. However, the DPSEEA is intended to be an inter-linking web rather than a straight chain for E&H problems, thereby demonstrating that for a number of driving forces multiple health effects may occur and these effects may be related to multiple exposures [26]. DPSEEA specifically includes stakeholders to assess these complex interactions between exposure and health effects making it particularly useful for identifying and monitoring environmental-health indicators and for designing cost effective interventions along the causal chain. DPSEEA has been adopted for: (i) monitoring health impacts of climate change in Europe [27]; (ii) developing E&H indicators to assess and monitor human health vulnerability, and (iii) measuring the effectiveness of climate change adaptation and mitigation [8]. Therefore, DPSEEA can provide the guidance necessary to develop an IEHM programme to measure and monitor the impact of environmental changes on human health.

The MEME framework (Additional file 3) used by the WHO, was designed to provide the conceptual basis for the development, collection and use of children’s E&H indicators [15,28] (Table 1). The framework describes the environmental health chain through the following components: exposure in different environmental settings leads to many different health outcomes (**Multiple effects**); and individual health outcome may be linked to many different exposures (**Multiple exposures**). Both exposures and health outcomes are affected by contextual conditions. Actions can be targeted at either the exposure or health outcome side, or at underlying contextual factors. The MEME is both a simplification and an extension of the DPSEEA. It combines the state of the environment, pressure and exposure components, recognizing that indicators of exposure may be assessed more or less directly, with state or pressure components serving as proxies for the actual exposure [29]. The MEME also emphasizes the complex relationships between environmental exposures and child health outcomes, specifically recognising the links between individual exposures and different health outcomes (Table 1). Additionally, it tries to encapsulate the concept that exposure, health, and associations between them, are affected by contextual factors, such as social, economic or demographic status [20]. The similarities of the MEME and DPSEEA mean that it is relatively simple to switch between them according to need [8].

Like the DPSEEA framework, an MEME approach could be applied to monitor and measure the impacts of environmental change on human health (Table 1). However, in practice, MEME has difficulty in distinguishing between the state of the environment and pressure on it. It does not separate proximal (exposure) from distal (pressure and state) causes, which nonetheless would be particularly useful for designing and applying interventions further up the causal chain [8,30]. Therefore, complex E&H issues are better described in DPSEEA than in MEME [8].

The IEHIA framework (Additional file 4) developed by the INTARESE project [31], was developed as a means of assessing health-related problems derived from the impact of policies related to E&H, and other interventions that affect the environment, taking into account the complexities, interdependencies, and uncertainties of the real world [8,31]. The IEHIA is a four-stage process, comprising: (1) Issue framing – defines the problem/purpose for assessment. This focuses and limits the scope of assessment and management options. (2) Design – the aim is to convert the conceptual model devised during issue framing into a detailed protocol for assessment. (3) Execution – the core of the assessment process. (4) Appraisal – synthesis and interpretation of results.

The IEHIA recognizes the concept of the DPSIR, DPSEEA and MEME [8,30]. It combines a qualitative approach for the selection and design of appropriate assessment methods, and a quantitative approach for carrying out integrated assessments of complex issues [31]. In practice, applying such a framework poses many challenges. The issue framing and design stage of the IEHIA require a strong transdisciplinary participation and involvement of multiple stakeholders with varying interests and levels of expertise. The execution and appraisal process involves modelling and analysis of complex, multivariate systems. Limited data availability and amplification of the uncertainties involved in devising and parameterising models presents further difficulties [32,33]. The main challenges of such an integrated approach are how to cope with the non-linearity of the natural processes, and how to describe the multi-causality of E&H issues inherent in most analyses [8,31].

The IEHIA approach could be applied to monitor and measure the impacts of environmental changes on human health [8] (Table 1). However, it is not specifically designed as a tool for developing an IEHM programme and does not explicitly identify or include entry points within the cause-effect chain for remedial actions [8].

### Monitoring programmes

Many national and international E&H monitoring programmes currently exist in Europe. We review eight E&H monitoring programmes: (1) the Arctic Monitoring and Assessment Programme (AMAP); (2) the European E&H Information System (ENHIS); (3) the German Environmental Survey (GerES); (4) the E&H Monitoring System in the Czech Republic (EHMS); (5) the PCBs Monitoring and Assessment Projects in Slovakia (PCBs in Slovakia); (6) the German Health Interview and Examination Survey for Children and Adolescents (KiGGS); (7) the Heat Wave Warning System in France (HWWS), and (8) the National Observatory of Climate Change Impact in France (ONERC). We concentrate on their use of an integrated methodology, i.e., the methods and tools for environmental health monitoring, for data linkage between environment and health endpoints, and for environmental health decisions-making support. In our assessment, we focus specifically on (i) aim/purpose; (ii) geographic scope; (iii) project duration; (iv) information about meta-data, and (v) data integration (Additional file 5) of the programmes, as these five properties determine their potential for supporting informed decision-making. We identify three types of monitoring programmes:

• International programmes, with the objective of documenting trends of pollution and comparing trends across countries, including a wide range of indicators, e.g., AMAP and ENHIS.

• National programmes, with the objective of documenting general health trends which focus on health based indicators, e.g., GerES, EHMS, PCBs in Slovakia and KiGGS.

• National programmes, with the objective of following up a specific risk, including both observation and forecasting, e.g., HWWS and ONERC.

The most common challenges that limit wider use of data from these programmes, beyond their scope and purpose, are:

**Knowledge limitations**: (i) narrow focus, such as targeting individual environmental stressors (e.g., PCBs in Slovakia), monitoring only one aspect of E&H problems, for example, monitoring the environment (e.g., GerES), or human health (e.g., KiGGS), but rarely integrating both. The AMAP focused on monitoring the levels of various pollutants in the Arctic (i.e., persistent organic contaminants, mercury, cadmium, lead, radioactivity, acidification and arctic haze, petroleum hydrocarbon pollution, stratospheric ozone depletion) and assessing the effects of pollution in several environmental compartments and the human population, yet was recently advised to improve integration in the context of climate together with related ecosystem or biodiversity [34]; (ii) complexity, uncertainty and lack of understanding of the underlying questions. Current monitoring programmes are mainly focused on priority pollutants (e.g., particulate matter (PM), nitrogen oxides (NOx), volatile organic compounds (VOCs), Polycyclic Aromatic Hydrocarbons (PAHs)), yet fail to rapidly include novel insights on emerging issues, such as climate change, waterborne stressors, ionizing and non-ionizing radioactivity [4,35], and (iii) lack of methods and tools to integrate data.

**Data issues**: (i) data availability. The ENHIS project found no data on air quality or bathing water quality for 43% of the EU population [36]; (ii) data sharing/accessibility. Due to privacy and confidentiality issues, human biomonitoring and health effects data are difficult to access, such as data from the PCBs projects in Slovakia and the EHMS project in the Czech Republic. The AMAP identified data access and sharing as key areas requiring improvement [34]; (iii) lack of harmonization between datasets. The ENHIS project found information gaps in harmonization and new data generation for E&H policies [37]; (iv) data geographical coverage. ENHIS has disclosed a limitation of the road network database, namely that it is missing big busy roads within the cities [36]. The PCBs projects in Slovakia did not provide sufficient data on the magnitude and hot-spots of PCBs-related health effects [38]. A human biomonitoring (HBM) subproject under the EHMS project only concerns urban and suburban populations in four selected regions. Improvement of the geographical coverage of existing monitoring programmes is needed [39].

**Low degree of interdisciplinary**: The programmes discussed above claim the involvement of a large number of disciplines; however, there are few monitoring programmes which are truly interdisciplinary [40]. For instance, the HWWS project in France only gathers inputs from two disciplines (meteorology and public health science) to assess the complex risks from heat waves and support decision-making.

**Language barrier**: one additional limiting issue of national environmental health monitoring programmes is that reports are often written only in the national language (e.g., the HWWS and ONERC programmes are only documented in French).

These gaps, together with data quality/data accuracy issues, have prevented a fuller integration of investigations of the health effects of environmental stressors at a European level, leading to overlapping efforts, and a lack of common meta-data development for policy decisions. Data sharing is not as simple as it would appear. Data collection is highly programme specific, and the nature of the data collected, the intended purpose of the data collection, and the level of ethical and intellectual property concerns are decided on a programme-by-programme level [41].

### Integrated environmental health monitoring: a conceptual framework

**Figure 1:**
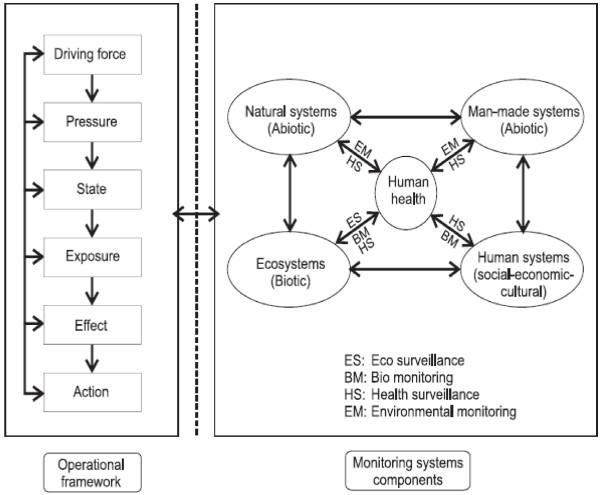
**A conceptual framework for integrated environmental health monitoring.** Driving force-Pressure-State-Exposure-Effect-Action (left box) describes the environmental health chain through the main components (see Additional file 2) that an integrated environmental health monitoring could follow as an operational framework. With human health at the core, the main components of an integrated environmental health monitoring framework (right box) are natural systems, man-made systems, ecosystems and human systems. The interconnections between these systems are environmental monitoring, bio-monitoring, eco-surveillance and health surveillance.

The need for integrated environmental health monitoring was recognized more than 10 years ago [31,42]. Yet, until now, there has been no concrete achievement [31,43]. The purpose of developing an IEHM framework (Figure 1) is to: (i) serve as a ‘think model’, so that users can apply it in a specific context and modify it according to their requirements, and (ii) provide a basis for further development of such a framework for a realistic IEHM programme.

An operative framework is important when developing and utilizing an IEHM programme. A systematic E&H approach based on such a framework facilitates the analysis of the whole natural-eco-anthropogenic system, or elements of this system [44,45]. The broad context of natural-eco-anthropogenic systems includes human health, cultural, social, economic, and political variables; and allows for more explicit links between the state of the environment and human welfare. Identification of these explicit links within the broader hazard surveillance category introduces the concept of surveillance of indirect hazards (e.g., biodiversity) [46,47].

In defining IEHM, we use the linear DPSEEA as an operational framework to identify the links between environment and health, and the entry points for action. We then extended this framework into four principles that capture how natural-eco-anthropogenic systems interact and how the linear DPSEEA framework and natural-eco-anthropogenic systems components are linked: (1) monitoring entire systems rather than unlinked individual components; (2) addressing the spatial-time dimensions of the system; (3) monitoring dynamic processes rather than static elements; and (4) designing methods and tools that would enable realising the goal of helping decision-making.

### Monitoring linear DPSEEA operational framework

The IEHM aims to improve our knowledge of causal links between E&H, as causality cannot be established by simply monitoring environmental factors and health outcomes simultaneously [48]. The pathways from source to exposure and to health effects are complex, and require additional information, e.g., on internal dose [49] or pharmacokinetic processes. In our IEHM framework (Figure 1), we combine DPSEEA (left part of the Figure 1) with natural-eco-anthropogenic systems (right part of the Figure 1). The IEHM framework addresses the needs that arise along the cause-effect diagram, and the fact that knowledge is imperfect, by monitoring a wide range of driving forces, pressures, states, exposure and health effect variables, and using this information to identify casual links, and help decision-making.

Central to this concept is exposure, which requires that people are present both at the place and at the time of a hazardous event. The concept of exposure is best developed in relation to pollutants in environmental media and the pollutant dose, i.e., the amount of the pollutant circulating in the human body as a result of assimilation, distribution, metabolism, and excretion (ADME) processes. From this perspective, biomarkers and human biomonitoring are necessary tools for the IEHM, as they are integrators of aggregate and/or cumulative exposure and effects [50].

Monitoring environmental quality is effective for an IEHM if it represent human exposure directly (e.g., noise) or indirectly (e.g., in drinking water via soil and ground water). Scientific research should clarify the relationships between environmental changes and their health impacts by documenting the relevant exposure pathways, their magnitudes, and by investigating exposure-dose–response relationships. Effective policies and measures reduce exposure and dose, both by improving environmental quality and by identifying populations from areas with poor quality of life and high exposure to pollution [51,52]. Understanding how exposures are embedded within the exposure-dose–response relationship is essential, particularly in an IEHM programme.

### Monitoring natural-eco-anthropogenic systems components

Human health is perceived as the integrated outcome of its ecological, socio-cultural, economic and institutional determinants at various spatial-temporal scales. It can be seen as a high-level integrated index that reflects the environmental state and, in the long-term, the sustainability of our natural and socio-economic environment [53]. Therefore, the IEHM framework (Figure 1) should be based on descriptions of the natural-eco-anthropogenic system rather than on its individual components.

The IEHM framework (Figure 1) includes four subsystems: natural, man-made, ecological, and human. The human system is incorporated into the IEHM framework as it is the most important factor in determining environmental changes [51]. With human health playing a central role, the IEHM addresses the interconnections between natural-eco-anthropogenic systems through four categories of monitoring (Figure 1): (i) environmental monitoring; (ii) eco-surveillance; (iii) biomonitoring; and (iv) health surveillance. The importance of linking environment monitoring and health surveillance with policy making has led to the addition of a fifth category of information: (v) the governance of policy intervention.

### Linking linear DPSEEA operational framework and natural-eco-anthropogenic systems components

The IEHM refers to simultaneous measurements of natural-eco-anthropogenic systems [51,53] and their health impacts over time at the same location with causative explanations [48-52]. This addresses the links between components of the monitoring systems and the E&H operational framework (Figure 1). In practice, the IEHM can be divided into a number of sub-programmes (e.g., environmental monitoring, eco-surveillance, bio-monitoring, health surveillance, etc.) which are linked by the use of the same parameters (monitoring systems components) and/or geographical location (E&H operational framework). The E&H operational framework and monitoring systems components connectivity in an IEHM programme can provide the web of causation within the complex real natural-eco-anthropogenic systems with human health, and help us to improve our knowledge on the relationships between changes of natural-eco-anthropogenic systems and human health. It views humans and natural-eco-anthropogenic systems as one interacting system. And while it is not necessarily just cause and effect, it is more than exposure and response in an IEHM programme.

### Keeping in mind the end goal of helping decision-making

In recent years, it has become apparent that many of the health risks facing society are systemic in nature – these are complex risks, set within wider social, economic and environmental contexts. Reflecting this, policy-making has become more wide-ranging in scope, more collaborative and more precautionary in approach [31,54]. Therefore, science needs first to anticipate, understand, assess, and reduce risks to human health and our environment to support governmental programmes to protect human health and safeguard the environment. This requires a consensus on the need to integrate data and an analytical methodology for monitoring and assessment [31,55]. The aim of an IEHM programme is to identify complex environmental health issues in a systematic and cause-effect chain approach to provide data useful for policy decisions on investments and resource allocation. However, the full integration and entire systems analyses in an IEHM may not necessarily be needed in all decision-making contexts. Particularly at strategic policy level such qualities are suitable, but at operational policy level specialized data or partial systems analyses can be more relevant.

### Integrated data from existing environmental health monitoring programmes

**Figure 2:**
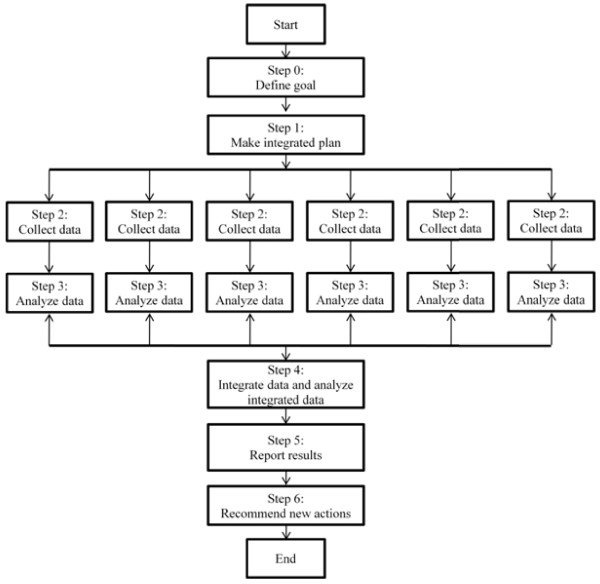
**Main steps of the integrated environmental health data from existing environmental health monitoring programmes.** This is a six steps work process. Step 0: define the goal of data integration. Step 1: make the integrated plan. Step 2: collect individual data. Step 3: analyse the individual data. Step 4: integrate the data and analyse the integrated data. Step 5: report results. Step 6: recommend new actions.

Instead of creating a completely new IEHM programme, a reasonable approach is to integrate data from existing E&H monitoring programmes. This approach is fully in line with the EU’s goal to make better use of existing environmental health data, for example with directives such as the Infrastructure for Spatial Information in the European Community (INSPIRE) [56]. However, this creates the challenge of how to integrate data from multiple monitoring programmes. Here, we first propose a structural work process (Figure 2) to: (i) overcome this challenge; (ii) create new ‘services’ based on the existing data, and (iii) identify data and knowledge gaps. This structural work process includes the following steps: Step 0: define the goal or ‘service’ of data integration; Step 1: set up an integrated plan that helps identify the required databases; Step 2: provide common access to collect meta-data from each individual database; Step 3: analyse data from each individual database and develop common meta-data information, including definition of data characteristics, format and process data, and assess data usefulness and quality; Step 4: retrieve and analyse integrated data; Step 5: statistical analysis, data presentation and report, and Step 6: recommendations. In order to link data from existing environmental health monitoring programmes, we need methodologies on data integration. Three data linking methods that are currently used in E&H fields are:

• Stochastic-mechanistic models for linking exposure and dose data, for example, the physiologically-based pharmacokinetic (PBPK) [57] and the biologically-based pharmacokinetic (BBPK) models [58]. PBPKs are powerful tools to link exposure to a parent compounds and/or active metabolites at the target sites of toxicity. BBPKs are increasingly used in risk assessment of environmental chemicals. In addition, other tools of hazard identification (e.g., Hazard analysis (HazAn), Hazard and operability (HazOp)) [59,60] and exposure assessment (e.g., Probabilistic risk assessment (PRA)) [60] can also be used to link exposure and dose data.

• Multiple empirical-statistical tools for linking dose and health effect data, including tools on dose–response assessment (e.g., biologically-based dose–response (BBDR) and mode-of-action (MOA)) [61,62] and risk characterization (e.g., Probabilistic exposure assessment (PEA), the Area under the curve (AUC) and the Joint probability curves (JPCs)) [60,63]. For instance, dose and risk calculation software (DCAL) [64] is comprehensive software for calculation of tissue dose and subsequent health risk from intake of pollutants or exposure to pollutants present in environmental media.

• Multiple systematic tools for linking exposure, dose and health effect data, such as Geographical Information Systems (GIS) [65], Bayesian belief networks (BBN) [66] and multiple lines and levels of evidence (MLLE) tools [67]. GIS links the indicators from environmental monitoring, biomonitoring and health surveillance in a visual way. These links might be represented as different layers where each layer holds data about a particular kind of health related environmental features. Each feature is linked to a position on the graphical image on a map and a record in an attributed table. Besides simply plotting environmental monitoring data and morbidity/mortality information on a map, GIS also offer important opportunities for interpolation or extrapolation of monitoring and modelling data [65]. MLLE is developed for epidemiological studies, human and ecological risk assessments [68,69]. MLLE as adapted by the US Natural Resource Management (NRM) [70] is mainly used to explore cause-effect relationships [71]. BBN is a probabilistic model that represents a set of random variables and their conditional interdependencies via a directed acyclic graph (DAG). For example, a BBN could represent the probabilistic relationships between diseases and symptoms. Given symptoms, the network can be used to compute the probabilities of various diseases. BBN is a method for the integration of the best possible data from a variety of sources [62,66].

Furthermore, as in any monitoring programme, efforts are needed to reduce uncertainties during data collection and data analysis. There are many sources of uncertainty in an IEHM programme which can generally be divided into two groups, quantitative uncertainty and qualitative uncertainty [33]. Quantitative uncertainty may derive from a lack of precision (e.g., variation in measurement due to insufficient number of observations, random sampling error) or a lack of accuracy (e.g., inaccuracies in observations, deriving from structural measurement errors, inappropriate extrapolation, confounding, etc.). Qualitative uncertainty indicates things that we do not know, but that cannot be captured in a statistical sense, e.g., how to quantify differences of opinion between scientists, differences in the framing of a problem, or inconsistencies in the scientific knowledge base? [33]. By linking to our structural work process in an IEHM programme, the quantitative uncertainty would be most likely restricted to step 2 to step 5 above, while qualitative uncertainties would be found at all steps.

All types of uncertainty require handling with appropriate techniques and there are a broad set of tools to do so. In summary, three types of techniques can be used for analysing uncertainties in the IEHM programmes: (i) Data quality assessment: Methods (e.g., Aguila tool [72], the Numerical, Unit, Spread, Assessment and Pedigree (NUSAP) system [73,74]) for a data quality assessment can be used to deal with the quantitative uncertainty, by evaluating whether data are fit for purpose. Such assessment involves “the scientific and statistical evaluation of data to determine whether they meet the objectives of the project, and thus are of the right type, quality, and quantity to support their intended use [75]”; (ii) Expert elicitation: this approach can be used to deal with qualitative uncertainty by consulting experts as a means to derive preliminary estimates for information about which scientific knowledge is as yet incomplete or inconsistent [33,75,76]; (iii) Methods based upon a typology of uncertainty: a typology of uncertainty can help to structure the different types of uncertainties (e.g., contextual uncertainty, model structure uncertainty, parameter uncertainty, input data uncertainty, etc.) [33,70]. This can in turn help to identify useful methods and techniques to deal with the uncertainties, ranging from stakeholder discussion to sensitivity and decision analyses [33,70]. Sensitivity and decision analyses can help to identify which sources of uncertainty mostly affect the final results [78-80] and the relative importance of each uncertain element. Once the major sources of uncertainty are known and prioritization is finished, suitable tools can be selected for further analysis. The uncertainty tool catalogue by Van der Sluijs et al. [81] provides guidance for selecting appropriate methods that match the characterization of the uncertainty in the typology. Refsgaard et al. [82] also describe various methods for dealing with uncertainties, and explain which purposes they may serve.

Finally, Quality assurance/Quality control (QA/QC) is one of the most critical components of an IEHM programme, and should make use of standard operating procedures (SOP) to provide data of known quality. The generation of reliable field and analytical data is best achieved through the development and implementation of a QA/QC plan [83,84]. Development of a rigorous QA/QC plan should be done in cooperation with all stakeholders throughout the entire monitoring period [85]. The fundamental elements of a QA/QC plan are: (1) **Data quality objectives** (DQOs) [86] are used to establish performance and acceptance criteria for field and laboratory measurement processes and set levels of acceptable measurement error. DQO is usually established for five aspects of data quality: representativeness, completeness, comparability, accuracy, and precision; (2) **Auditing**: Undertaking regular audits of field, laboratory and data management operations provides valuable feedback on the adequacy, implementation, and effectiveness of existing quality systems. Regular quality audits allow for the information gained to be used to make improvements to the quality system or plan and provide a benchmark for maintaining a level of competence; (3) **Data generation and acquisition**: In accordance with established DQOs, the generation and acquisition of high-quality monitoring data relies on the adherence to quality control measures during field sampling operations and laboratory analyses. Typically, quality control is maintained during field work operations by adhering to standardized sampling protocols, analysis of QA/QC samples, and the regular calibration and maintenance of field instrumentation; (4) **Data validation and usability**: The quality review and validation of data can be readily undertaken by assessing all data for compliance with the project’s DQOs. Quality checks are also undertaken along all data flow paths, particularly at data entry steps. If data are found to fall outside the accepted DQO limits, then corrective actions (e.g., re-analyse suspect samples, re-sampling and reanalysis, accept data with an acknowledged level of bias and imprecision, discard data, etc.) may be undertaken; and (5) Data Management: Data quality through the various data generation, acquisition, assessment and storage processes should be managed by a series of quality control measures. These can include managing the chain of custody; defined data flow paths, the use of standard field data sheets and laboratory reports, and information management (database) systems.

In addition, QA/QA and uncertainties are closely linked. For example, QA/QC requirements can improve accuracy and reduce uncertainty, while in turn, reducing uncertainty can improve data of known quality. In practice, the assessment of uncertainty and QA/QC may need to be implemented in parallel, e.g., laboratories should report an estimate of the uncertainties associated with each measurement.

## Discussion

The approaches to IEHM for IEHIA outlined in this paper combine two main components: an approach for designing and carrying out a realistic IEHM programme of complex, systematic E&H problems (IEHM conceptual framework); and a qualitative approach for integrated data from existing multiple monitoring programmes (IEHM structural work process, data integration-uncertainty-QA/QC methods). Neither of these approaches is without its limitations and challenges. The former relate to the design/operation challenges of an IEHM programme; the latter implies the ability to integrate data from multiple monitoring sources.

### Challenges

Following the IEHM framework and IEHM work process, a realistic IEHM programme may need to integrate existing monitoring programmes. This may generate a constraint on the harmonizing measurement techniques [52]. For instance, many of the standard design rules and methods used in the establishment of a new IEHM programme cannot be easily applied since existing E&H monitoring programmes often have specific monitoring objectives with different measurement protocols and sampling designs. These constraints on the form and function of an IEHM programme may compromise and complicate some key design issues, but in some respects this makes it even more important that design issues are fully considered at the onset [52].

In the design process, the expectations of end-users also need to be considered [52]. Unlike purely research-orientated activities, an IEHM programme should also provide input data for policy makers and other stakeholders. In this context, partial information from an IEHM programme may not be relevant if policy needs require information to be valid at higher levels of integration and harmonization. Data from an IEHM programme must therefore be defensible against criticism, e.g., representativeness, precision, consistency and reproducibility. These goals can be achieved by thorough planning and early stakeholders’ participation in all the steps of the IEHM design process [52].

Combining the DPSEEA (cause-effect approach) with natural-eco-anthropogenic systems (systematic approach), an IEHM programme has to be highly interdisciplinary and must therefore be designed taking into account the priorities, perspective and expertise of stakeholders at different levels. When support for decisions-making is needed, the issues underlying operational choices should be understandable to their intended audience, which could be a significant challenge, but could be achieved by the ‘analytical-deliberative’ approach of the National Research Council in the USA [13] and the extended peer community approach [87,88]. In addition, the procedure that includes a diversity of actors for selection of hot-spots for human biomonitoring research in Belgium developed by Keune et al. (2010) [89], could also be considered to develop and inform the programme design. Given the inherent complexity of E&H problems, it may be one of the first disciplines to benefit from the current paradigm shift from multi-disciplinary science to transdisciplinary science to tackle complex societal issues.

In addition, current data from E&H monitoring programmes face many challenges: (i) fragmentation of datasets and sources; (ii) lack of harmonization between datasets at different geographical scales [90], and (iii) issues of data quality and accuracy.

In practice, using an IEHM work process to access data presents a number of challenges [63,91]: (i) obtaining data from other agencies is difficult, and in many cases impossible; (ii) legal and the level of ethical [38] restrictions prevent access to a particular dataset; (iii) it is difficult to obtain the cooperation of agency hierarchy, who decide whether or not to participate in data sharing; (iv) data sharing requires compatibility between different computer systems as well as the availability of information system personnel; (v) data integration requires the cooperation of system administrators, directors of programmes, and services consumers, and (vi) data integration is costly and time consuming, and information overload are also barriers to data integration across multiple organizations.

Additionally, there are a number of technical challenges concerning data analysis: (i) increases in data volume; (ii) increasing need for interdisciplinary use of data; and (iii) integration of data among systems to answer questions that address diverse societal benefits [92].

### Development needs

Globally, action such as the Global Monitoring for Environment and Security (GMES) programme [93] are a way forward to improve the interoperability and integration of data. In Europe, following the EU’s Environment and Health Action Plan (EHAP), there are several ongoing international ‘harmonization actions’, e.g., the INSPIRE Directive, the Consortium to Perform Human Bio-monitoring on a European Scale (COPHES) [94], the European Health Examination Survey (EHES) [95], the EU Menu [96], etc. There are similar ongoing projects in countries outside the EU, e.g., the Environmental Public Health Tracking/Surveillance in Canada [97], and the National Environmental Public Health Tracking Programme in the USA [98]. As discussed above, there are many challenges to integrate existing E&H data across the EU, therefore, there is still a need for establishing either a IEHM programme at the EU level or multiple IEHM programmes at individual national level, that follow the common standards and share the same goals, to: (i) improve data availability and utility; (ii) develop the strategy on IEHM and the methodology for integrating data from multiple monitoring programmes; (iii) promote knowledge translation for everyday use and for policy making, and (iv) facilitate integration of the health effects of environmental stressors at a European level.

## Conclusions

We support the notions that there is a need to establish an IEHM programme either at the EU level or at individual national level but following common standards, including systematic techniques for integrating data, assessing uncertainty and strengthening QA/QC. Based upon the review of frameworks and monitoring programmes in the areas of environmental health, and the identification of the key elements and qualities essential for an IEHM programme, we propose the following definition of IEHM: ‘IEHM is a systemic process to measure, analyse, interpret state and changes of natural-eco-anthropogenic systems over time at the same location with causative explanations across compartments along the cause-effect chain’.

By applying DPSEEA as a operational framework to identify the links between environment and health, we develop a conceptual IEHM framework to monitor and measure the impacts of environmental changes on human health with the following characteristics: (i) monitoring entire systems instead of their individual components; (ii) addressing the spatial and time dimensions; (iii) monitoring causal chain processes instead of having static elements, and (iv) keeping in mind the end goal of helping decision-making.

The IEHM work process demonstrates the steps needed to integrate data from multiple monitoring sources. It can facilitate achieving the IEHIA goals of greater efficiency, quality and better-informed decisions in ways that support specific information management needs.

## Abbreviations

AMAP: Arctic Monitoring and Assessment Programme; AUC: Area Under the Curve; ADME: Assimilation, Distribution, Metabolism, and Excretion; BBN: Bayesian Belief Networks; BBDR: Biologically-Based Dose–response; BBPK: Biologically-Based Pharmacokinetic; COPHES: Consortium to Perform Human Bio-monitoring on a European Scale; DQO: Data Quality Objectives; DAG: Directed Acyclic Graph; DCAL: Dose and Risk Calculation Software; DPSEEA: Driving force-Pressure-State-Exposure-Effect-Action; DPSIR: Driving forces-Pressures-State of the environment-Impacts-Responses; DSR: Driving force-State-Response; E&H: Environment and health; EHAP: Environment and Health Action Plan; EHMS: Environmental Health Monitoring System in the Czech Republic; ECEH: European Centre for Environment and Health; EEA: European Environmental Agency; ENHIS: European Environment and Health Information System; EHES: European Health Examination Survey; ECHI: European initiatives European Community Health Indicators; EIEHMRS: European Integrated Environment and Health Monitoring and Response System; EU: European Union; HazAn: Hazard analysis; HazOp: Hazard and operability; HWWS: Heat Wave Warning System in France; HBM: Human Bio-Monitoring; GIS: Geographical Information Systems; GerES: German Environmental Survey; KiGGS: German Health Interview and Examination Survey for Children and Adolescents; GMES: Global Monitoring for Environment and Security; INSPIRE: Infrastructure for Spatial Information in the European Community; INTARESE: Integrated Assessment of Health Risks of Environmental Stressors in Europe; IEHIA: Integrated Environmental Health Impact Assessment; IEHM: Integrated Environmental Health Monitoring; ISO: International Organization for Standardization; JPCs: Joint Probability Curves; MOA: Mode-Of-Action; MEME: Multiple Exposures Multiple Effects; MLLE: Multiple Lines and Levels of Evidence; NUSAP: Numerical, Unit, Spread, Assessment and Pedigree, ONERC, National Observatory of Climate Change Impact in France; NOx: Nitrogen Oxides; PM: Particulate Matter; PBPK: Physiologically-Based Pharmacokinetic; PAHs: Polycyclic Aromatic Hydrocarbons; PCBs: Polychlorinated Biphenyls; PSR: Pressure-State-Response; PEA: Probabilistic Exposure Assessment; PRA: Probabilistic Risk Assessment; QA: Quality Assurance; QC: Quality Control; SOP: Standard Operating Procedures; UNEP: United Nations Environmental Programme (UNEP); EPA: United States Environmental Protection Agency; NRM: United States Natural Resource Management; VOCs: Volatile Organic Compounds; WHO: World Health Organisation (WHO).

## Competing interests

The authors declare they have no competing interests.

## Authors’ contributions

HYL planned this work and wrote the manuscript. HYL, AB, RS, MD and ES contributed to the concept development and implementation. AB was the project leader. All authors approved the final version.

## Supplementary Material

Additional file 1 **DPSIR (Driving forces-Pressures-State -Impacts-Responses) framework (source: EEA).** For the purpose and the key elements of the DPSIR framework, see text under section Frameworks. Click here for file

Additional file 2 **DPSEEA (Driving force-Pressure-State-Exposure-Effect-Action) framework (source: WHO).** For the purpose and the key elements of the DPSEEA framework, see text under section Frameworks. Click here for file

Additional file 3 **MEME (Multiple Exposures-Multiple Effects) framework (source: WHO).** For the purpose and the key elements of the MEME framework, see the text under section Frameworks. Click here for file

Additional file 4 **IEHIA (Integrated Environmental Health Impact Assessment) framework (source: INTARESE).** For the purpose and the key elements of the IEHIA framework, see the text under section Frameworks. Click here for file

Additional file 5 **Overview of eight monitoring programmes with their aim, monitoring location, period, data information and integrated methodologies.** For acronyms please see the text. Click here for file
