# Supplementary material for: Approaches to integrated monitoring for environmental health impact assessment
Source: Environ Health. 2012 Nov 21;11:88. doi: 10.1186/1476-069X-11-88 (PMC3526392; doi:10.1186/1476-069X-11-88)
Supplement: Additional file 1 — DPSIR (Driving forces-Pressures-State -Impacts-Responses) framework (source: EEA). For the purpose and the key elements of the DPSIR framework, see text under section Frameworks. [file 1476-069X-11-88-S1.docx]

## Supplementary file 1 – DPSIR (Driving forces-Pressures-State -Impacts-Responses) framework (source: EEA). For the purpose and the key elements of the DPSIR framework, see text under section Frameworks.
