# Supplementary material for: Approaches to integrated monitoring for environmental health impact assessment
Source: Environ Health. 2012 Nov 21;11:88. doi: 10.1186/1476-069X-11-88 (PMC3526392; doi:10.1186/1476-069X-11-88)
Supplement: Additional file 2 — DPSEEA (Driving force-Pressure-State-Exposure-Effect-Action) framework (source: WHO). For the purpose and the key elements of the DPSEEA framework, see text under section Frameworks. [file 1476-069X-11-88-S2.docx]

## Supplementary file 2 – DPSEEA (Driving force-Pressure-State-Exposure-Effect-Action) framework (source: WHO). For the purpose and the key elements of the DPSEEA framework, see text under section Frameeorks.
