# Supplementary material for: Approaches to integrated monitoring for environmental health impact assessment
Source: Environ Health. 2012 Nov 21;11:88. doi: 10.1186/1476-069X-11-88 (PMC3526392; doi:10.1186/1476-069X-11-88)
Supplement: Additional file 3 — MEME (Multiple Exposures-Multiple Effects) framework (source: WHO). For the purpose and the key elements of the MEME framework, see the text under section Frameworks. [file 1476-069X-11-88-S3.docx]

Supplementary file 3 – MEME (Multiple Exposures-Multiple Effects) framework (source: WHO). For the purpose and the key elements of the MEME framework, see the text under section Frameworks.
