# Supplementary material for: Approaches to integrated monitoring for environmental health impact assessment
Source: Environ Health. 2012 Nov 21;11:88. doi: 10.1186/1476-069X-11-88 (PMC3526392; doi:10.1186/1476-069X-11-88)
Supplement: Additional file 4 — IEHIA (Integrated Environmental Health Impact Assessment) framework (source: INTARESE). For the purpose and the key elements of the IEHIA framework, see the text under section Frameworks. [file 1476-069X-11-88-S4.docx]

## Supplementary file 4 – IEHIA (Integrated Environmental Health Impact Assessment) framework (source: INTARESE). For the purpose and the key elements of the IEHIA framework, see the text under section Frameworks.
