# Supplementary material for: Approaches to integrated monitoring for environmental health impact assessment
Source: Environ Health. 2012 Nov 21;11:88. doi: 10.1186/1476-069X-11-88 (PMC3526392; doi:10.1186/1476-069X-11-88)
Supplement: Additional file 5 — Overview of eight monitoring programmes with their aim, monitoring location, period, data information and integrated methodologies. For acronyms please see the text. [file 1476-069X-11-88-S5.pdf]

Supplement file 5 - Overview of eight monitoring programmes with their aim, monitoring location, period, data information and integrated methodologies. For acronyms please see the text.

Project acronym	Aim	Location	Period	Data information	Integrated methodologies
**AMAP**	To provide reliable and sufficient information on the status of, and threats to, the arctic environment.<br>To provide scientific advice on actions to be taken in order to support arctic governments in their efforts to take remedial and preventive actions relating to contaminants.	The terrestrial and marine areas, north of the arctic circle	1991-2012	Environment<br>Atmospheric contaminants<br>Marine contaminants<br>Radioactivity<br>Freshwater and terrestrial contaminants<br>Health<br>UV radiation and climate change	Guideline and methodology were developed for each monitoring system, quality control and general monitoring issues
**ENHIS**	To monitor the environment and health situation and trends in the countries in the European region and to evaluate the effectiveness of policies.<br>To conduct comparisons between countries on the basis of targets set in European-wide action programmes.<br>To regular reporting on environment and health to support decision makers and also to provide information to professionals and the general public.<br>To exchange information, data, knowledge and good practice examples in the field.	Europe	2008-	Environment<br>Air quality<br>Food safety<br>Chemical safety<br>Water and sanitation<br>Mobility and transport<br>Housing<br>UV and ionizing radiation<br>Occupational hazards<br>Health<br>Exposure of population to environmental stressors	Methodology was developed for thirty indicators giving the rationale, definitions, required data elements, calculation methods, data sources, interpretations and policy-relevance.
**EHMS**	To provide high quality background data for decision-making by the national and local authorities in the fields of health care policy, health risks management and control, and environmental protection.	Czech Republic	1994-2006	Environments (136 contaminant factors)<br>Air pollution<br>Drinking water pollution<br>Noise<br>Soil contamination<br>Health<br>Dietary exposure and human bio-monitoring	Methodology was developed for monitored factors and indicators and their limits, information system and data processing, and QA/QC system

Supplement file 5 (Cont.)

Project acronym	Aim	Location	Period	Data information	Integrated methodologies
**GerES**	To generate, update, and evaluate representative data in order to facilitate an environmental health related observation and reporting of information at the national level.	East-, West-Germany	1985-2006	Environment<br>Domestic environment: tap water, dust deposit, content of vacuum cleaner bag and indoor air.<br>Community: water works sample and dust fall outdoors.<br>Health<br>Human bio-monitoring, diet and personal air	Methodology was developed for fieldwork, experimental chemical analysis, and data analysis (including checking and revising data, matching different data files, weighting).
**KiGSS**	To improve the information available on the health of the up-and-coming generation in Germany and to fill gaps in knowledge.	East-, West-Germany	1990-1992<br>2003-2006	Health (1990-1992, 4730 participants; 2003 -2006, 17,641 participants)<br>Measure: physical and mental health<br>Questionnaire: health status, health behaviour, health care utilization, social and migrant status, living conditions<br>Environment<br>Environmental determinants of health.	Methodology was developed for the participants interviews, physical examinations, blood and urine samples, and data processing
**ONERC**	To collect and disseminate information, studies and researches on the risks linked to climate change, to formulate recommendations for action and prevention.	France	2001	Climate change (15 indicators)<br>Different sources<br>Several datasets<br>Population data<br>Exposure of population to climate risk	Report on specific themes, e.g. human health, relying on the indicators.
**PCB in Slovakia**	To assess environmental PCB exposure of population, with the main focus on prenatal and early postnatal exposure of infants.<br>To evaluate effects of PCB exposure on health status of adult and children population, mainly disruption of endocrine and immune systems and neurobehavioral and developmental alterations.	Michalovce and Svidnik/Stropkov regions, Eastern Slovakia	2001-	Pollutants<br>PCBs and toxic metals.<br>Health (8 indicators)<br>Thyroid gland, glucose homeostasis and neurodevelopment disorders	Report on specific themes, e.g. human health, relying on the indicators.
**HWWS**	To identify heat waves that presents a potential danger to public health and to orientate management measures.	France	2003-	Environmental variables<br>Temperature and air quality (O_3, PM_{10})<br>Health<br>Mortality<br>Morbidity	Analysis of the temperature data, including the probability of being above threshold<br>If the probability are medium to high, analysis of additional risk factors<br>During a heat wave or immediately after, analysis of the health data to orientate the actions.
